# Association of a Novel IgG3 Allele With Malaria in Children From the Sepik Region of Papua New Guinea

**DOI:** 10.1093/infdis/jiaf390

**Published:** 2025-07-25

**Authors:** Maria Saeed, Elizabeth H Aitken, Myo T Naung, Caitlin Bourke, Kenneth W Wu, Rhea J Longley, Amy W Chung, Timon Damelang, Benson Kiniboro, Ivo Mueller, Stephen J Rogerson

**Affiliations:** Department of Infectious Diseases, The Peter Doherty Institute for Infection and Immunity, University of Melbourne, Melbourne, Victoria, Australia; Department of Infectious Diseases, The Peter Doherty Institute for Infection and Immunity, University of Melbourne, Melbourne, Victoria, Australia; Department of Microbiology and Immunology, The Peter Doherty Institute for Infection and Immunity, University of Melbourne, Melbourne, Victoria, Australia; Infection and Global Health Division, Walter and Eliza Hall Institute, Parkville, Victoria, Australia; Infection and Global Health Division, Walter and Eliza Hall Institute, Parkville, Victoria, Australia; Infection and Global Health Division, Walter and Eliza Hall Institute, Parkville, Victoria, Australia; Infection and Global Health Division, Walter and Eliza Hall Institute, Parkville, Victoria, Australia; Department of Medical Biology, University of Melbourne, Melbourne, Victoria, Australia; Faculty of Tropical Medicine, Mahidol University, Bangkok, Thailand; Department of Microbiology and Immunology, The Peter Doherty Institute for Infection and Immunity, University of Melbourne, Melbourne, Victoria, Australia; Department of Infectious Diseases, The Peter Doherty Institute for Infection and Immunity, University of Melbourne, Melbourne, Victoria, Australia; Department of Microbiology and Immunology, The Peter Doherty Institute for Infection and Immunity, University of Melbourne, Melbourne, Victoria, Australia; Vector Borne Disease Unit, Papua New Guinea Institute of Medical Research, Goroka, Papua New Guinea; Infection and Global Health Division, Walter and Eliza Hall Institute, Parkville, Victoria, Australia; Department of Medical Biology, University of Melbourne, Melbourne, Victoria, Australia; Department of Infectious Diseases, The Peter Doherty Institute for Infection and Immunity, University of Melbourne, Melbourne, Victoria, Australia; Department of Medicine, The Peter Doherty Institute for Infection and Immunity, University of Melbourne, Melbourne, Victoria, Australia

**Keywords:** IgG3 polymorphism, malaria, *P falciparum*, *P vivax*, antibody

## Abstract

**Background:**

Susceptibility to malaria can be influenced by host genetic factors, including immune response genes. Antibodies against *Plasmodium* antigens are known to play an important role in protection from clinical disease. Polymorphisms in these antibodies may result in different functional properties that could provide protection from malaria.

**Methods:**

Immunoglobulin G1 (IgG1) and immunoglobulin G3 (IgG3) alleles and IgG3 hinge region were investigated by polymerase chain reaction and Sanger sequencing in a longitudinal cohort of children aged 1–3 years (N = 203) from the East Sepik region of Papua New Guinea. Linear regression was used to investigate associations between immunoglobulin alleles and *Plasmodium* infections.

**Results:**

Seventy-eight percent of the children were either heterozygous (n = 82 [40%]) or homozygous (n = 77 [38%]) for IGHG3*30 (G3m29), a novel IgG3 allele. G3m29 has a long hinge region of 4 exons. Significantly fewer *Plasmodium* spp infections were observed in children with the IGHG3*30 allele compared to children without the allele (β = −1.736 [95% confidence interval {CI}, −3.39, −.079]; *P* = .038). This effect was most noticeable for *Plasmodium vivax* asymptomatic infections as IGHG3*30 carriers had on average 1 fewer infection in the 18-month follow-up period compared with non-IGHG3*30 allele carriers (β = −1.06 [95% CI, −2.01, −.12]; *P* = .028). Additionally, IGHG3*30 allele carriers had significantly lower levels of IgG to *P vivax* vaccine candidate proteins compared to non-IGHG3*30 allele carriers.

**Conclusions:**

The IGHG3*30 allele is highly prevalent in the East Sepik region and is associated with fewer *Plasmodium* spp infections.

## Teaser

A novel IgG3 allele (IGHG3*30) is highly prevalent in the East Sepik region of Papua New Guinea. IGHG3*30 is protective against *Plasmodium* spp infections, particularly *P vivax* asymptomatic infections, with a possible mechanism being antibody-dependent phagocytosis.

Malaria is a life-threatening disease affecting millions of people worldwide. In 2023, there were 597 000 malaria-related deaths worldwide, mostly in children <5 years of age [1]. Malaria has unarguably altered the human genome and has been a driving force behind evolutionary selection [2]. Red blood cell polymorphisms such as sickle cell anemia, thalassemia, glucose-6-phosphate dehydrogenase deficiency, and ovalocytosis are reported to be protective against severe malaria and are widely distributed in malaria-endemic regions [3]. Acquisition of malaria-specific antibodies is associated with protection from clinical malaria [4, 5], and antibody features like Fc-mediated effector functions have the potential to control malaria [6, 7].

Antibody allotypes on the heavy chain of immunoglobulin G (IgG) 1, IgG2, IgG3, and IgG4 constant region are written as G1m, G2m, G3m, and G4m, respectively. IgG3 is the most polymorphic subclass with 15 reported allotypes, mostly occurring in the C_H_2 and C_H_3 domains [8, 9]. These polymorphisms may have structural and/or functional consequences, including changes in length of the hinge region, antibody half-life, and Fc-mediated effector functions [9, 10]. IgG3 allotypes G3m15* and G3m16* encoded by IGHG3*17, IGHG3*18, IGHG3*19, IGHG3*22, and IGHG3*23 have arginine to histidine substitutions at the 435 position (p. Arg435His) in the C_H_3 domain, which are associated with increased transplacental IgG3 transfer [10–12], and infants of G3m15* and/or G3m16* allele-carrying mothers have less clinical malaria [12]. Some IgG3 allotypes alter binding affinities for effector molecules, such as Fcγ receptors (FcγRs) or C1q, which can influence effector functions such as antibody-dependent cellular phagocytosis (ADCP), cellular cytotoxicity, and complement activation [13–17]. Therefore, it is imperative to investigate the significance of G3m allotypes in malaria.

Recently a novel IgG3 allele, hereafter referred to as IGHG3*30 (G3m29), was reported in a cohort of pregnant women from the Sepik region in Papua New Guinea (PNG) [18]. IGHG3*30 is characterized by a novel substitution of histidine to glutamine at the 433 position (p. His433Glu) in the C_H_3 domain [18]. This position has not been described as a polymorphic position before. Functional amino acid substitutions include Pro291Leu, Ser384Asp, Arg435His, and Phe436Tyr [18]. IGHG3*30 also has an Arg392Lys substitution whose functional significance is unclear [19]. IGHG3*30 has altered functions and interactions with FcγRs. Plasma from women with the IGHG3*30 allele was able to induce significantly higher levels of ADCP but not significantly different antibody-dependent neutrophil phagocytosis (ADNP) of *Plasmodium falciparum*–infected erythrocytes compared to plasma from non-IGHG3*30 allele–carrying women. Moreover, monoclonal antibodies made with IGHG3*30 heavy chains showed increased affinity to FcγRIIa and significantly higher ADCP of *P falciparum* VAR2CSA DBL3-coated beads and 2-fold higher ADNP of *P falciparum*–infected erythrocytes as compared to other Gm allotypes [18].

Here we examine the prevalence and potential protective role of IGHG3*30 allele, in a cohort of 203 young PNG children followed longitudinally for *Plasmodium* spp infection and febrile illness over a period of 18 months [20]. The G1m and G3m allotypes of the children were determined and associations with number of malaria infections were examined. We also measured the length of the hinge region and compared levels of IgG against a panel of *Plasmodium vivax* antigens in children with and without the IGHG3*30 allele.

## MATERIALS AND METHODS

### Study Design and Sample Collection

**Figure 1.**
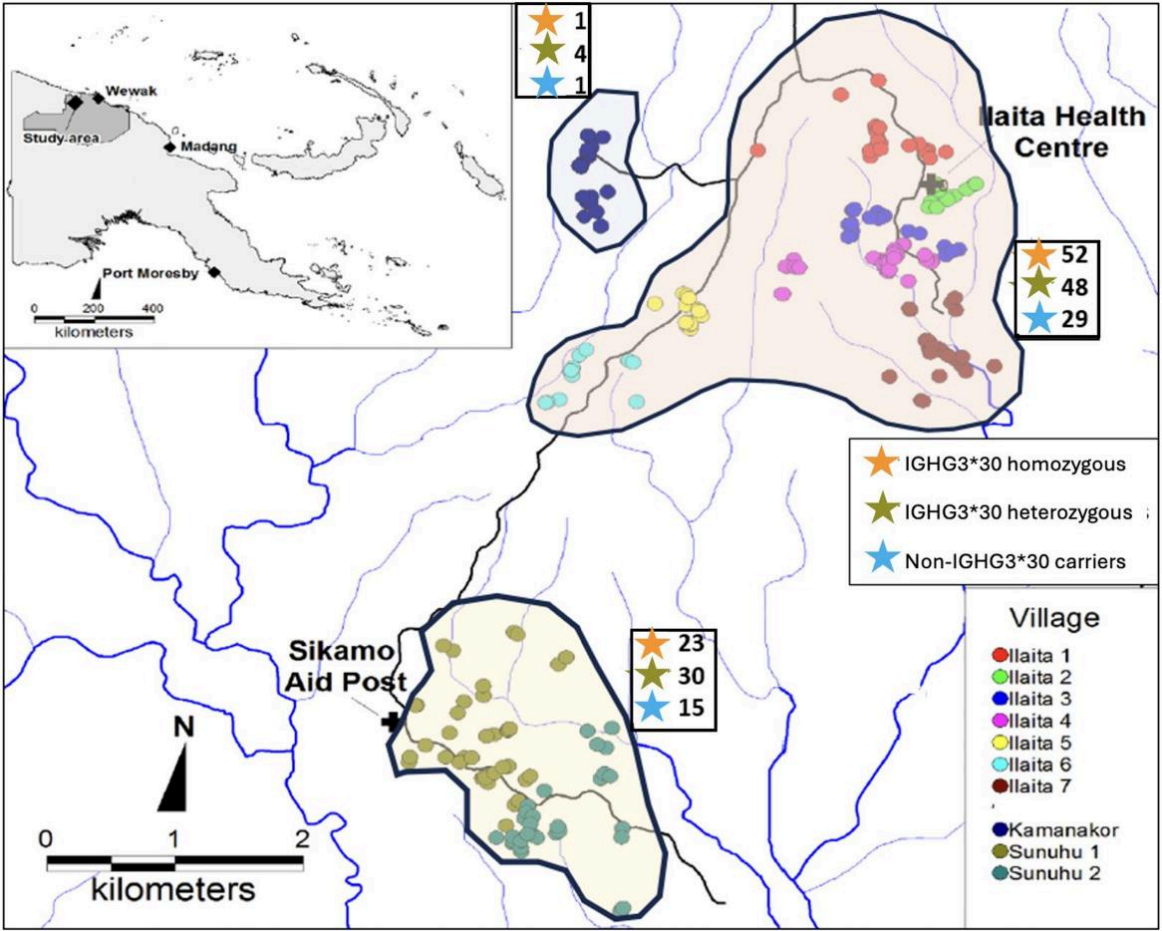
Map of study area. Adapted from Lin et al, 2010 [20]. Dots represent the participant's houses, crosses represent health centers, black lines represent roads, and blue lines represent rivers and streams. Stars represent the number of homozygous IGHG3*30 allele carriers (top), heterozygous IGHG3*30 allele carriers (middle), and non-IGHG3*30 allele carriers (bottom).

The cohort details and sample collection protocols have been described previously [20]. In brief, in a longitudinal study, 264 PNG children aged 1–3 years from 11 villages in the Illaita area of Maprik District, East Sepik Province, PNG (Figure 1), were recruited in March 2006 and actively followed up for 18 months for *Plasmodium* spp infection and febrile illness [20]. *Plasmodium* spp infection was confirmed by light microscopy and polymerase chain reaction (PCR) [21]. DNA extracted from blood samples (N = 203) was available for this study to investigate IgG1 and IgG3 alleles and their association with *Plasmodium* spp infections. Baseline characteristics of the study participants are shown in Table 1.

**Table 1.** Baseline Characteristics of Study Participants Based on IgG3 Allele

Characteristics	Total (N = 203)	IGHG3*30 (n = 159)^a^	Non-IGHG3*30 (n = 44)^b^	*P* Value
Age, mo, mean ± SD	23.5 ± 8.2	22.9 ± 8.1	25.4 ± 8.3	.074
Hemoglobin, g/dL, mean ± SD	9.0 ± 0.95	9.0 ± 0.98	9.0 ± 0.82	.967
Sex				
Male	117 (57.63)	94 (59.12)	23 (52.27)	.416
Female	86 (42.36)	65 (40.88)	21 (47.72)	.416
Village				
Ilaita 1	20 (9.85)	15 (9.43)	5 (11.36)	.703
Ilaita 2	12 (5.91)	9 (5.66)	3 (6.82)	.725
Ilaita 3	20 (9.85)	15 (9.43)	5 (11.36)	.703
Ilaita 4	27 (13.30)	23 (14.46)	4 (9.09)	.456
Ilaita 5	13 (6.40)	9 (5.66)	4 (9.09)	.484
Ilaita 6	12 (5.91)	11 (6.92)	1 (2.27)	.468
Ilaita 7	25 (12.31)	18 (11.32)	7 (15.91)	.412
Kamanakor	6 (2.95)	5 (3.14)	1 (2.27)	.999
Sunuhu 1	43 (21.18)	33 (20.75)	10 (22.73)	.778
Sunuhu 2	26 (12.81)	21 (13.20)	5 (11.36)	.746
Total No. of *Plasmodium* spp infections, mean **±** SD	12.5 ± 4.95	12.1 ± 5.1	13.9 ± 4.3	**.029**

Data are presented as No. of participants (%) unless otherwise indicated. Means were compared by Mann-Whitney *U* test while percentages were compared by Pearson χ^2^/Fisher exact test. *P* values <.05 are bold.

Abbreviation: SD, standard deviation.

^a^IGHG3*30 includes homozygous IGHG3*30 allele carriers (n = 77) and heterozygous IGHG3*30 allele carriers (n = 82).

^b^Non-IGHG3*30 includes homozygous IGHG3*01 (G3m5*) (n = 5), homozygous IGHG3*14 (G3m21*) (n = 35), and heterozygous IGHG3*01*/IGHG3*14* (n = 4) individuals.

### Definition of Categories

*Plasmodium* spp infections were identified and categorized (Supplementary Table 1) [20–22]. The summary of *Plasmodium* spp infections in children is shown in Supplementary Table 2.

#### Ethics Approval

Ethical approval was obtained from institutional review boards of the PNG Medical Research Advisory Committee (approval 05.19), University Hospitals Case Medical Center (Cleveland, Ohio, USA), and the Swiss Tropical Institute. Written informed consent was obtained from parent(s) or guardian(s) before recruitment. The Walter and Eliza Hall Institute of Medical Research human research ethics committee (07/07) provided the ethical approval for samples to be used for antibody investigations.

### Polymerase Chain Reaction

Genomic DNA was enriched using Illustra GenomiPhi V2 DNA amplification kit, Cytiva (GE Healthcare Life Sciences, Australia) as per the manufacturer's guidelines. PCR was optimized to amplify the genes encoding C_H_2 and C_H_3 domains of IgG3 [23], IgG1-C_H_1 [24], and IgG1-C_H_3 domains [25] and genes encoding IgG3 hinge [26]. Primer sequences are provided in Supplementary Table 3.

For the C_H_2 and C_H_3 domains of IgG3, 30- μL PCR reactions were prepared containing 2–5 ng of enriched DNA, 1X AccuPrime PCR buffer II, 0.6 μL of 100% dimethyl sulfoxide, 1.2 μL of each primer (0.5 μM), and 0.15 μL of AccuPrime Taq polymerase (Thermo Fisher). For the C_H_1 and C_H_3 domains of IgG1, 30-μL PCR reactions were prepared containing 2–5 ng of enriched DNA, 1X AccuPrime PCR buffer II, 0.6 μL of each primer (0.5 μM), and 0.15 μL of AccuPrime Taq polymerase. PCR conditions for the 2 reactions included 94°C for 30 seconds (initial denaturation), 94°C for 30 seconds (denaturation), 60°C for 30 seconds (annealing), 68°C for 1 minute (extension), and 35 cycles of amplification followed by 72°C for 7 minutes (final extension) on a BioRad T100 Thermal Cycler [18]. For the IgG3 hinge region, 25 μL PCR reaction was prepared containing 2–5 ng of enriched DNA sample, 1X HotStar buffer, 1 μL of each primer (1 μM), and 1.25 U of HotStarTaq polymerase (QIAGEN). HotStarTaq DNA polymerase was activated at 95°C for 15 minutes, then 38 cycles of 95°C for 30 seconds, 61°C for 30 seconds, and 72°C for 30 seconds, and final extension at 72°C for 7 minutes were performed [26].

PCR products were visualized on a 2% agarose gel in 1X TAE buffer at 110 V for 1 hour (Supplementary Figure 1). For identification of single-nucleotide polymorphisms, PCR products were submitted to the Australian Genome Research Facility (Victorian Comprehensive Cancer Centre, Parkville) for Sanger sequencing. Sequences were analyzed in Geneious Prime software (version 2024.0.5). For hinge length estimation, band sizes of 806, 618, and 430 bp corresponding to long (L), medium (M), and small (S) hinge phenotypes were identified.

#### Total IgG Levels to *P vivax* Proteins

Six *P vivax* antigens (fam-a_1, fam-a_2, RBP2b_1 [reticulocyte binding protein 2b], RBP2b_2, PTEX150 [*Plasmodium* translocon of exported proteins], and MSP5 [merozoite surface protein 5]) were selected because they were previously described as top serological markers of exposure [27]. There is high level of genetic diversity in *Plasmodium* antigens, so 2 commonly found haplotypes of *P vivax* fam-a and RBP2b were included. Proteins were coupled to BioRad COOH magnetic beads (BioRad, California, USA) according to the manufacturer's guidelines. Plasma samples collected at enrollment from study participants were diluted 1:100 and added to coupled beads to measure total IgG using multiplex Luminex system as previously described [27]. Raw mean fluorescence intensities were converted to relative antibody units using a 5-parameter logistic regression standard curve generated from the hyperimmune positive control plasma pool from PNG using RshinyAPP (https://shaziaruybal.shinyapps.io/COVIDClassifyR/).

### Data Analysis

Data were analyzed using Stata17 and Prism version 10 (GraphPad Software). Associations between IGHG3*30 and *Plasmodium* spp infections were measured via linear regression. Associations between G1m and G3m allotypes were investigated using χ^2^ test. Differences in the antibody levels between individuals with and without IGHG3*30 were assessed by the nonparametric Mann-Whitney *U* test and Kruskal-Wallis test. Multiple linear regression was performed to assess the associations between IGHG3*30 allele carrier status and *Plasmodium* spp infections after adjusting for IgG levels to 6 *P vivax* proteins.

## RESULTS

### Novel IGHG3*30 Allele Is Highly Prevalent in Papua New Guinean Children From Sepik

**Figure 2.**
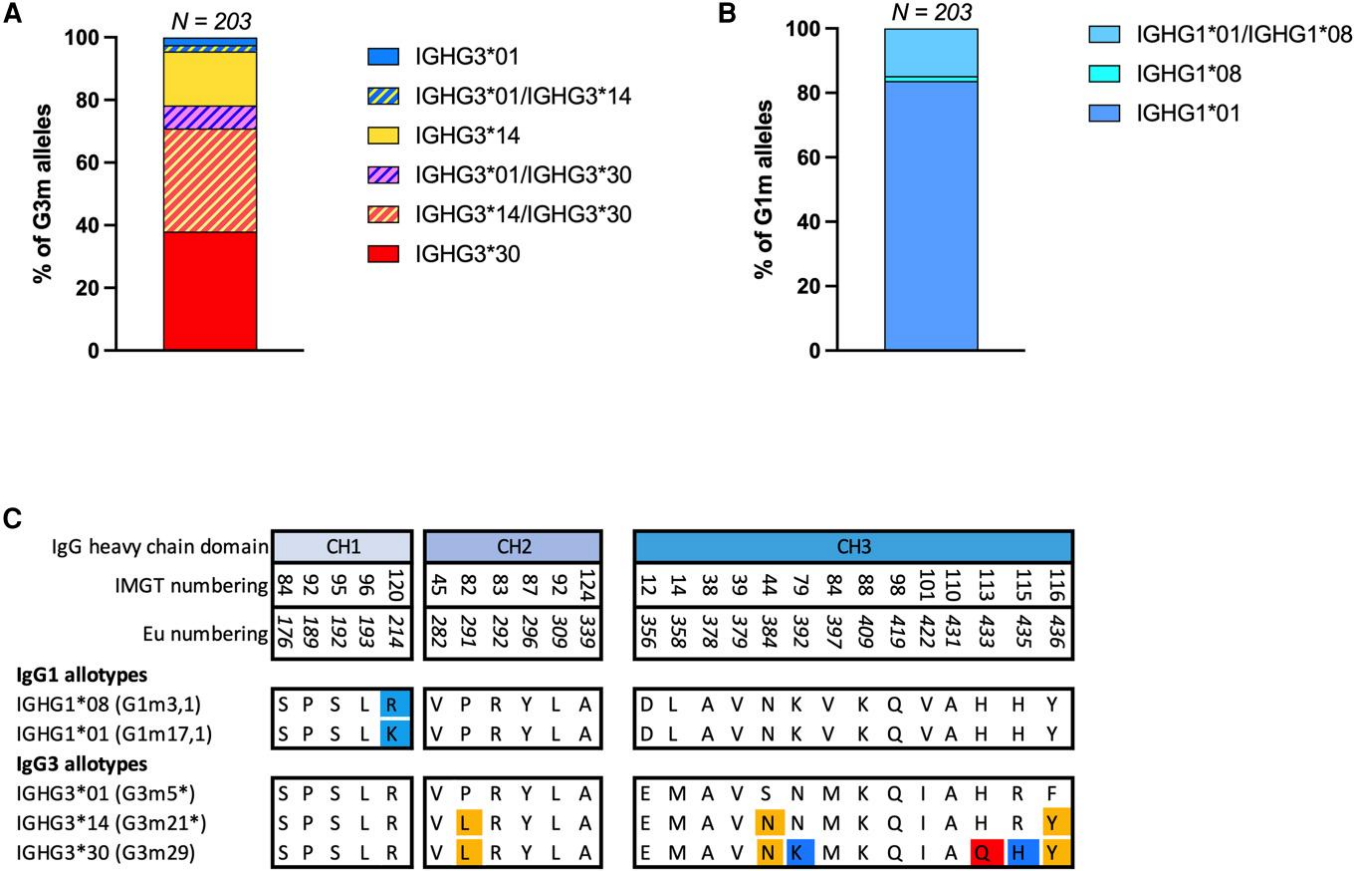
IgG1 and IgG3 polymorphisms identified in children. Percentage of G3m alleles (*A*) and G1m alleles (*B*) in children. *C*, Highlighted amino acids correspond to the polymorphic positions in the heavy chain constant region of IgG1 and IgG3 allotypes. Abbreviations: Eu, European Union; IMGT, international ImMunoGeneTics information system®.

IgG3 polymorphisms of 203 PNG children from the Sepik region were determined (Table 2). Two previously described G3m allotypes were identified; IGHG3*01 (G3m5*) and IGHG3*14 (G3m21*) (Figure 2) [9]. The IGHG3*30 allele was highly prevalent, with 78% of the children being either homozygous or heterozygous for IGHG3*30 (Figure 2*A*).

**Table 2.** Number of Children With Specific Genotype and Hinge Length Polymorphism

Allele	No. of Children (N = 203)	LL (806 bp)	SS (430 bp)	LS (806/430 bp)
IGHG3 allele				
IGHG3*01	5	5	…	…
IGHG3*14	35	32	3	…
IGHG3*30	77	77	…	…
IGHG3*01/IGHG3*14	4	4	…	…
IGHG3*01/IGHG3*30	15	15	…	…
IGHG3*14/IGHG3*30	67	64	…	3
IGHG1 allele		…	…	…
IGHG1*01	170	…	…	…
IGHG1*01/IGHG1*08	30	…	…	…
IGHG1*08	3	…	…	…

Abbreviations: LL, long hinge region; LS, heterozygous; SS, short hinge region.

A PCR-based method was used to identify IgG3 hinge length polymorphisms. Based on amplicon size on agarose gel electrophoresis (Supplementary Figure 1*D*), children's genotypes were long (LL) (n = 197/203), short (SS) (n = 3/203), and LS heterozygous (n = 3/203); medium hinge length (MM) was not present. Majority of the children carrying an IGHG3*30 allele (n = 156/159) were homozygous for long hinge. Most of the IGHG3*14 children had long hinge (n = 100/106) but 3 IGHG3*14 children were homozygous for small hinge (n = 3/106), and 3 children (IGHG3*14*/IGHG3*30) were heterozygous “LS” (Table 2).

Two previously described IGHG1 alleles were also identified in the cohort: IGHG1*01 (G1m17,1) and IGHG1*08 (G1m3,1) (Figure 2*B*) [9]. Most children were homozygous for IGHG1*01 (n = 170), 30 were heterozygous for IGHG1*01/IGHG1*08, and only 3 were IGHG1*08 homozygous (Table 2).

### Children Heterozygous for IGHG3*30 Have Fewer *P vivax* Asymptomatic Infections

To investigate whether IGHG3*30 is protective, the association between the presence of IGHG3*30 allele and *Plasmodium* spp infections was evaluated. IGHG3*30 allele carriers had on average 1.7 fewer *Plasmodium* spp infections in the follow-up period compared to non-IGHG3*30 allele carriers (β = −1.74 [95% confidence interval {CI}, −3.39, −.08]; *P* = .038). Notably, IGHG3*30 allele carriers had 1 fewer *P vivax* asymptomatic infection compared to non-IGHG3*30 carriers (β = −1.06 [95% CI, −2.01, −.12]; *P* = .028). The general trend was for less *Plasmodium* spp infection and morbidity in IGHG3*30 carriers compared to non-IGHG3*30 (Table 3).

**Table 3.** Association of IGHG*30 Allele Carriage With *Plasmodium* spp Infections Between IGHG3*30 Carriers and Non-IGHG3*30 Carriers (N = 203)

Infection	Coefficient	(95% CI)	*P* Value
*Plasmodium* **spp** infections	−1.73	(−3.39, −.08)	**.038**
Asymptomatic *Plasmodium* **spp** infections	−1.60	(−2.89, −.46)	**.007**
*P vivax* asymptomatic infections	−1.06	(−2.01, −.12)	**.028**
*P falciparum* asymptomatic infections	−0.19	(−1.04, .63)	.640
Symptomatic *Plasmodium* **spp** infections	−0.49	(−1.32, .34)	.246
*P vivax* symptomatic infections	0.03	(−0.63, .70)	.915
*P falciparum* symptomatic infections	−0.50	(−1.09, .08)	.094
*P vivax* severe malaria infections	−0.09	(−.20, .02)	.115
*P vivax* severe + *P falciparum* hyperparasitemia	−0.09	(−.22, .03)	.125

Non-IGHG3*30 was reference category for each analysis. Numbers in bold are significant, *P* < .05.

Abbreviation: CI, confidence interval.

To investigate the gene dosage effect of IGHG3*30, children were grouped as IGHG3*30-homozygous, IGHG3*30-heterozygous, or non-IGHG3*30 carriers. Children heterozygous for IGHG3*30 allele had on average 2.3 fewer *Plasmodium* spp infections in the follow-up period compared to non-IGHG3*30 allele carriers (β = −2.34 [95% CI, −4.13, −.54]; *P* = .011). Most notably, children heterozygous for IGHG3*30 had 1.4 fewer *P vivax* asymptomatic infections in the follow-up period compared to non-IGHG3*30 carriers (β = −1.47 [95% CI, −2.49, −.44]; *P* = .005). No similar association was seen for children homozygous for the IGHG3*30 allele (Supplementary Table 4).

To investigate the association of IGHG1*01 with malaria, children were grouped as IGHG1*01-homozygous and IGHG1*01-heterozygous. Children heterozygous for IGHG1*01 had on average 2 fewer *Plasmodium* spp infections in the follow-up period compared to children homozygous for IGHG1*01 (β = −1.95 [95% CI, −3.88, −.02]; *P* = .047); however, no significant associations were observed for specific *Plasmodium* infections (Supplementary Table 5).

### Children Heterozygous for Gm Haplotype IGHG1*01, IGHG3*30 Have Fewer *Plasmodium* spp Infections

There was a statistically significant linkage between IGHG3*30 and IGHG1*01 (χ^2^ = 8.807, *P* = .012; Supplementary Table 6). Since IgG alleles are often inherited in combinations as haplotypes, IGHG3*30 and IGHG1*01 were grouped together as a haplotype to further investigate any association between Gm haplotypes and *Plasmodium* spp infections. Children with the Gm IGHG1*01, IGHG3*30 heterozygous haplotype had on average almost 3 fewer *Plasmodium* spp infections (β = −2.95 [95% CI, −5.35, −.55]; *P* = .016), compared to non-IGHG1*01, IGHG3*30 children (grouped together as “other haplotypes”). This effect was most significant for *P vivax* asymptomatic infections; children with the Gm IGHG1*01, IGHG3*30 heterozygous haplotype had on average 1.4 fewer infections (β = −1.4 [95% CI, −2.81, −.05]; *P* = .042) (Table 4).

**Table 4.** Association Between Gm Haplotypes and *Plasmodium* spp Infections (N = 203)

Infection	Coefficient	(95% CI)	*P* Value
*Plasmodium* spp infections			
Gm IGHG1*01, IGHG3*30 heterozygous	−2.95	(−5.35, −.55)	**.016**
Gm IGHG1*01, IGHG3*30 homozygous	−0.14	(−1.61, 1.31)	.842
Asymptomatic *Plasmodium* spp infections			
Gm IGHG1*01, IGHG3*30 heterozygous	−2.387	(−4.15, −.061)	**.009**
Gm IGHG1*01, IGHG3*30 homozygous	−0.37	(−1.45, .71)	.496
*P vivax* asymptomatic infections			
Gm IGHG1*01, IGHG3*30 heterozygous	−1.43	(2.81, −.05)	**.042**
Gm IGHG1*01, IGHG3*30 homozygous	−0.05	(−.89, .79)	.901
*P falciparum* asymptomatic infections			
Gm IGHG1*01, IGHG3*30 heterozygous	−0.65	(−1.87, .56)	.291
Gm IGHG1*01, IGHG3*30 homozygous	0.08	(−.65, .83)	.815
Symptomatic *Plasmodium* spp infections			
Gm IGHG1*01, IGHG3*30 heterozygous	−0.62	(−1.84, .58)	.308
Gm IGHG1*01, IGHG3*30 homozygous	−0.17	(−.91, .57)	.650
*P vivax* symptomatic infections			
Gm IGHG1*01, IGHG3*30 heterozygous	0.20	(−.76, 1.17)	.677
Gm IGHG1*01, IGHG3*30 homozygous	0.34	(−.24, .93)	.246
*P falciparum* symptomatic infections			
Gm IGHG1*01, IGHG3*30 heterozygous	−0.79	(−1.64, .05)	.065
Gm IGHG1*01, IGHG3*30 homozygous	−0.52	(−1.04, −.00)	**.048**
*P vivax* severe malaria infections			
Gm IGHG1*01, IGHG3*30 heterozygous	−0.14	(−.31, .02)	.094
Gm IGHG1*01, IGHG3*30 homozygous	0.01	(−.08, .11)	.796
*P vivax* severe + *P falciparum* hyperparasitemia			
Gm IGHG1*01, IGHG3*30 heterozygous	−0.124	(−.31, .060)	.187
Gm IGHG1*01, IGHG3*30 homozygous	0.020	(−.092, .13)	.725

Other haplotypes are used as reference category for all comparisons. Numbers in bold are significant, *P* < .05.

Abbreviation: CI, confidence interval.

By contrast, the number of *Plasmodium* spp infections in children with the Gm IGHG1*01, IGHG3*30 homozygous haplotype was similar to those with the other haplotypes (β = −.15 [95% CI, −1.61, 1.32]; *P* = .842). The general trend was for less disease in children heterozygous for Gm IGHG1*01, IGHG3*30 compared to other haplotypes, and no difference between children homozygous for Gm IGHG1*01, IGHG3*30 compared to other haplotypes. The exception to this was that children who were Gm IGHG1*01, IGHG3*30 heterozygous (β = −.79 [95% CI, −1.65, .05]; *P* = .065) or homozygous (β = −.52 [95% CI, −1.04, −.004]; *P* = .048) had trends toward fewer *P falciparum* symptomatic infections compared to other haplotypes (Table 4). We assessed the associations between the novel allele and *P falciparum* force of infection in IGHG3*30 carriers and noncarriers and found no significant association (*P* = .43).

### Children With IGHG3*30 Have Lower Levels of Total IgG to *P vivax* Proteins

**Figure 3.**
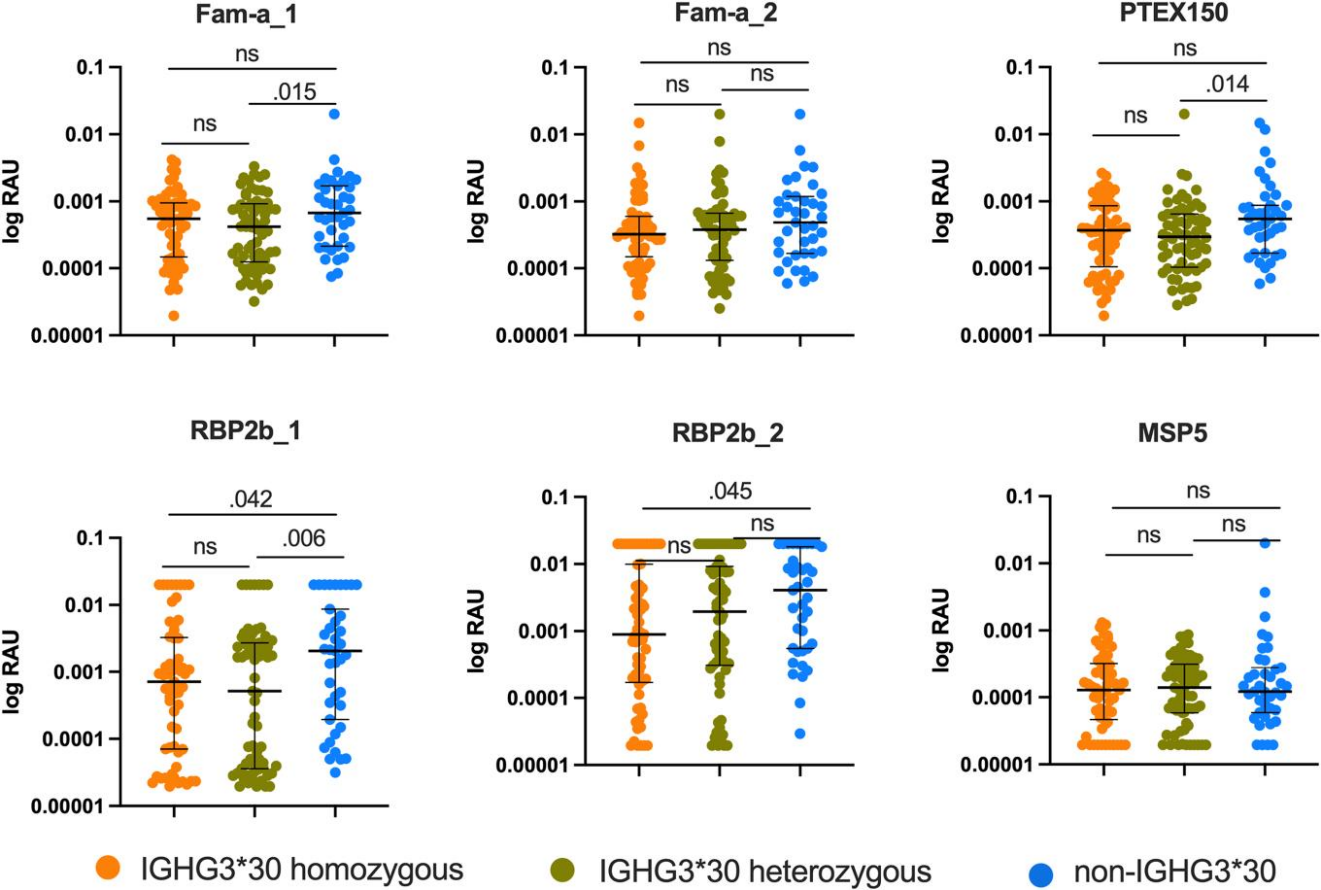
Total IgG levels to *Plasmodium vivax* proteins in IGHG3*30 homozygous, IGHG3*30 heterozygous, and non-IGHG3*30 carriers. Data are presented as median and interquartile range. The y-axis shows log-transformed relative antibody unit (RAU) values. *P* values were determined by Mann-Whitney *U* test; Abbreviation: ns; nonsignificant.

Previous studies investigating IgG allotypes have shown conflicting data in terms of allotype impact on IgG levels and subclass distribution [18, 28]. To understand at a high level any potential differences in IgG acquisition and how this may impact the association with reduced infections in IGHG3*30 allele carriers, we measured IgG to 6 *P vivax* recombinant proteins in children with available plasma samples at enrollment (n = 167). Overall, IGHG3*30 allele carriers had significantly less IgG to 3 of 6 *P vivax* antigens (Supplementary Figure 2). Notably, children heterozygous for IGHG3*30 had significantly less IgG to the same 3 proteins, whereas children homozygous for IGHG3*30 had significantly less IgG only to RBP2b_1 and RBP2b_2 (Figure 3). We grouped children into tertiles (low, medium, high) based on combined antibody levels to the 6 proteins and adjusted the regression model accordingly. We found that IGHG3*30 carriers had significantly fewer *P vivax* asymptomatic infections after adjusting for IgG levels (β = −.29 [95% CI, −1.20, .62]; *P* = .035) (Supplementary Table 7).

## DISCUSSION

Here, we confirmed the presence and high prevalence of the novel IgG3 allele, IGHG3*30, in a cohort of children from East Sepik region of PNG. The high prevalence of IGHG3*30 allele suggests a protective effect due to possible positive selection in an area of PNG with high malaria transmission. IgG3 variants with H435 (also included in IGHG3*30) are common in malaria-endemic African countries. Histidine in this position promotes IgG3 binding to FcRn, resulting in a longer half-life and greater transplacental transfer, which has been associated with protection from malaria [12]. However, the effects of IGHG3*30 on protective effector functions are less clear [18]. Additional studies on IGHG3*30 allele in other malaria-endemic regions are needed to clarify the potential selective pressures driving IGHG3*30 to a high prevalence and its role in malaria outcomes.

Similar proportions of children were heterozygous and homozygous for the IGHG3*30 allele, which is not following Hardy-Weinberg equilibrium (HWE). This could be due to the evolutionary forces like natural selection modulating the allelic distribution in this population [29]. The deviation from HWE in our study population is consistent with our earlier observations in pregnant women [18]. In both cases, we observed an unexpectedly high frequency of IGHG3*30 homozygotes. This pattern may indicate the action of nonneutral evolutionary forces. In our earlier study, we described functional features of IGHG3*30, such as stronger binding to FcγRIIa H131 and enhanced ADCP of *P falciparum*–infected erythrocytes, that could confer a selective advantage [18]. These immune benefits could align with the allele's association with reduced risk of asymptomatic *P vivax* infections we observed. In addition to positive selection, demographic factors could contribute. IGHG3*30 is most common in East Sepik, a geographically isolated region with a complex migration history. Gene flow and potential founder effects in this population may explain the observed enrichment of the allele and the deviation from HWE.

Importantly, children with the IGHG3*30 allele had fewer *Plasmodium* spp infections, particularly *P vivax* asymptomatic infections, as compared to children without IGHG3*30. This effect was particularly evident for children heterozygous for IGHG3*30. Asymptomatic infections not only serve as a reservoir for continued malaria transmission and pose challenges for malaria elimination, but they can also progress to severe disease [30]. The strong association with *P vivax* asymptomatic infections may reflect their high incidence in this cohort. Children in this cohort acquired immunity to *P vivax* infections more quickly than to *P falciparum* [20].

It is not clear why IGHG3*30 is more protective in heterozygous form, but several factors might explain this observation. First, in our earlier study, IGHG3*30 homozygotes had lower total and antigen-specific IgG3 levels but showed enhanced ADCP via increased FcγRIIa binding. It is possible that protection peaks in heterozygotes due to a balance between expression levels and effector function, while homozygotes might experience diminishing returns or altered subclass switching. Second, some form of balancing selection could be occurring. Co-inheritance of IGHG3*30 with IGHG1*01, for example, may contribute to immune modulation that differs between heterozygotes and homozygotes. Last, the difference in cohorts may also play a role. The current study focused on young children, whereas our previous work involved pregnant women. Age, immune status, and exposure history could influence the functional impact of this allele. Moreover, the distinct B-cell populations in heterozygotes undergo allelic exclusion and are committed to expressing a single allele. Thus, distinct B cells produce IgG3 with His433 and Glu433, which may result in an additive effect.

Several studies have examined the associations between IgG allotypes and malaria immunity [28, 31, 32]. The distribution of IgG allotypes differs among populations and has been associated with malaria resistance or susceptibility [28]. A study reported that Beninese children with IGHG3*01 had significantly higher ADCP of merozoites compared to those with other IgG3 allotypes [32]. Certain IgG allotypes mediate increased opsonic phagocytosis, reflecting their increased affinity to one or the other FcγR [33, 34]. Previously, monoclonal antibodies with the IGHG3*30 sequence showed high affinity to FcγRIIa [18], which is present on classical monocytes and neutrophils, and women with the IGHG3*30 allele showed greater antibody-dependent phagocytosis of infected erythrocytes. Given the importance of FcγRIIa in antibody-dependent phagocytosis [35, 36], we postulate that enhanced ADCP could contribute to the observed protective association. FcγRIIa-mediated phagocytosis by monocytes and neutrophils has been linked to protection in several malaria studies, and the increased ADCP we observed in IGHG3*30 carriers aligns well with this mechanism. However, other immune pathways are involved in protection from blood-stage infection. For example, FcγRIIIa-mediated ADCC or complement activation, both of which are less efficient with IGHG3*30, may play a more prominent role in protection from severe or symptomatic disease. Our current data suggest that IGHG3*30 may enhance one pathway, ADCP, while having a more limited effect on others.

A long hinge region increases IgG3 flexibility and increases opsonic phagocytosis [37]. In Ghana, a medium hinge length was associated with greater odds of cerebral malaria compared to a long hinge length [26]. All the children homozygous for the IGHG3*30 allele (n = 77) in our study had a long IgG3 hinge region (Supplementary Table 2). Notably, we also found that most children with IGHG3*14 (n = 32/35) had a long IgG3 hinge, while 3 had a small hinge and 3 were heterozygous (LS) (Table 2). To our knowledge this is the first study to report this novel hinge length polymorphism in IGHG3*14. Previously, 2 IgG3 variants IGHG3*01 (long hinge) and IGHG3*04 (small hinge) under G3m5* mosaic were shown to have the same sequence and to vary in hinge length [16].

IgG allotypes could also affect susceptibility to malaria and disease outcomes by influencing the subclass distribution and affinity of antimalarial antibodies [38–41]. Damelang et al reported elevated proportions of IgG1 (measured as % of total IgG) but lower proportions of IgG3 in plasma of women with IGHG3*30 allele as compared to women with IGHG3*01 allele [18]. Caucasian individuals with the Gm 3,23;5,13 phenotype showed lower concentrations of IgG1 but higher concentrations of IgG3 in normal human serum [40]. In the present study, levels of IgG to *P vivax* proteins, previously defined as serological markers of exposure [27], were assessed. IgG levels were significantly lower in children heterozygous for IGHG3*30 compared to children without IGHG3*30. Further research is needed to understand the impact of the IGHG3*30 allele on the acquired immune response to infection, and to assess whether antibodies isolated from these children have different abilities to induce effector functions. As acquisition and boosting of antibodies is impacted by exposure to *Plasmodium* parasites, and levels of antibodies can change over time, further work is needed to understand the potentially complex interplay of these factors and the IGHG3*30 itself.

Our study corroborates the discovery of IGHG3*30 in PNG [18] and extends the finding beyond pregnant women. In this longitudinal study we established relationships between IGHG3*30 allele and *Plasmodium* spp infections. A longitudinal study has an advantage over a cross-sectional design such as getting information at multiple time-points, but limitations of our study include the sample size and unequal distribution of Gm allotypes between groups, which affected statistical power. Future studies could investigate antigen-specific subclasses and antibody half-life, to better characterize IGHG3*30. With few severe malaria cases, associations of IGHG3*30 allele with severe malaria could not be made. Further studies of IgG3 alleles in other malaria-endemic regions and globally could help elucidate if IGHG3*30 is found more broadly and should examine potential associations between IGHG3*30 and severe malaria. Including control antigens like tetanus or influenza could help to show if IgG levels for those antigens follow a different trend as compared to *Plasmodium* antigens.

In conclusion, our data provide compelling evidence that the IGHG3*30 allele confers protection against *Plasmodium* spp infections and is probably under positive selection. Its high prevalence in children from the East Sepik region of PNG underscores its potential functional significance. The strong associations between IGHG3*30 and lower antibody levels against *P vivax* antigens further suggest that malaria has been a key evolutionary driver in maintaining this polymorphism within the population.

## Supplementary Material

jiaf390_Supplementary_Data

## Supplementary Data

Supplementary materials are available at *The Journal of Infectious Diseases* online (http://jid.oxfordjournals.org/). Supplementary materials consist of data provided by the author that are published to benefit the reader. The posted materials are not copyedited. The contents of all supplementary data are the sole responsibility of the authors. Questions or messages regarding errors should be addressed to the author.

## References

[1] World Health Organization . World malaria report 2024. 2024. https://www.who.int/teams/global-malaria-programme. Accessed 7 August 2025.

[2] Carter R, Mendis KN. Evolutionary and historical aspects of the burden of malaria. Clin Microbiol Rev 2002; 15:564–94. 12364370 10.1128/CMR.15.4.564-594.2002 PMC126857

[3] Kariuki SN, Williams TN. Human genetics and malaria resistance. Hum Genet 2020; 139:801–11. 32130487 10.1007/s00439-020-02142-6 PMC7271956

[4] Cohen S, McGregor IA, Carrington S. Gamma-globulin and acquired immunity to human malaria. Nature 1961; 192:733–7. 13880318 10.1038/192733a0

[5] Weaver R, Reiling L, Feng G, et al The association between naturally acquired IgG subclass specific antibodies to the PfRH5 invasion complex and protection from *Plasmodium falciparum* malaria. Sci Rep 2016; 6:33094. 27604417 10.1038/srep33094 PMC5015043

[6] Aitken EH, Damelang T, Ortega AP, et al Developing a multivariate prediction model of antibody features associated with protection of malaria-infected pregnant women from placental malaria. Elife 2021; 10:e65776. 34181872 10.7554/eLife.65776 PMC8241440

[7] Opi DH, Kurtovic L, Chan JA, Horton JL, Feng G, Beeson JG. Multi-functional antibody profiling for malaria vaccine development and evaluation. Expert Rev Vaccines 2021; 20:1257–72. 34530671 10.1080/14760584.2021.1981864

[8] Damelang T, Brinkhaus M, van Osch TL, et al Impact of structural modifications of IgG antibodies on effector functions. Front Immunol 2024; 14:1304365. 38259472 10.3389/fimmu.2023.1304365 PMC10800522

[9] Lefranc MP, Lefranc G. Human Gm, Km, and Am allotypes and their molecular characterization: a remarkable demonstration of polymorphism. Methods Mol Biol 2012; 882:630–80. 10.1007/978-1-61779-842-9_34 22665258

[10] Stapleton NM, Andersen JT, Stemerding AM, et al Competition for FcRn-mediated transport gives rise to short half-life of human IgG3 and offers therapeutic potential. Nat Commun 2011; 2:599. 22186895 10.1038/ncomms1608 PMC3247843

[11] Einarsdottir H, Ji Y, Visser R, et al H435-containing immunoglobulin G3 allotypes are transported efficiently across the human placenta: implications for alloantibody-mediated diseases of the newborn. Transfusion (Paris) 2014; 54:665–71. 10.1111/trf.12334 23829325

[12] Dechavanne C, Dechavanne S, Sadissou I, et al Associations between an IgG3 polymorphism in the binding domain for FcRn, transplacental transfer of malaria-specific IgG3, and protection against *Plasmodium falciparum* malaria during infancy: a birth cohort study in Benin. PLoS Med 2017; 14:e1002403. 28991911 10.1371/journal.pmed.1002403 PMC5633139

[13] Vidarsson G, Dekkers G, Rispens T. IgG subclasses and allotypes: from structure to effector functions. Front Immunol 2014; 5:520. 25368619 10.3389/fimmu.2014.00520 PMC4202688

[14] Richardson SI, Lambson BE, Crowley AR, et al IgG3 enhances neutralization potency and Fc effector function of an HIV V2-specific broadly neutralizing antibody. PLoS Pathog 2019; 15:1008064. 10.1371/journal.ppat.1008064 PMC6936867 31841557

[15] Damelang T, Rogerson SJ, Kent SJ, Chung AW. Role of IgG3 in infectious diseases. Trends Immunol 2019; 40:197–211. 30745265 10.1016/j.it.2019.01.005

[16] de Taeye SW, Bentlage AEH, Mebius MM, et al Fcγr binding and ADCC activity of human IgG allotypes. Front Immunol 2020; 11:740. 32435243 10.3389/fimmu.2020.00740 PMC7218058

[17] Crowley AR, Richardson SI, Tuyishime M, et al Functional consequences of allotypic polymorphisms in human immunoglobulin G subclasses. Immunogenetics 2022; 75:1–16. 35904629 10.1007/s00251-022-01272-7 PMC9845132

[18] Damelang T, Kassa MW, Lopez E, et al Novel IgG3 allotype identified in women from malaria endemic regions that modulates Fc effector functions. SSRN [Preprint]. Posted online 1 December 2022. doi:10.2139/ssrn.4287904

[19] Rispens T, Davies AM, Heer PO, et al Dynamics of inter-heavy chain interactions in human immunoglobulin G (IgG) subclasses studied by kinetic Fab arm exchange. Biol Chem 2014; 289:6098–109. 10.1074/jbc.M113.541813 PMC3937676 24425871

[20] Lin E, Kiniboro B, Gray L, et al Differential patterns of infection and disease with *P. falciparum* and *P. vivax* in young Papua New Guinean children. PLoS One 2010; 5:9047. 10.1371/journal.pone.0009047 PMC2816213 20140220

[21] McNamara DT, Thomson JM, Kasehagen LJ, Zimmerman PA. Development of a multiplex PCR-ligase detection reaction assay for diagnosis of infection by the four parasite species causing malaria in humans. J Clin Microbiol 2004; 42:2403–10. 15184411 10.1128/JCM.42.6.2403-2410.2004 PMC427836

[22] Manning L, Laman M, Law I, et al Features and prognosis of severe malaria caused by *Plasmodium falciparum*, *Plasmodium vivax* and mixed *Plasmodium* species in Papua New Guinean children. PLoS One 2011; 6:29203. 10.1371/journal.pone.0029203 PMC3245265 22216212

[23] Dambrun M, Dechavanne C, Emmanuel A, et al Human immunoglobulin heavy gamma chain polymorphisms: molecular confirmation of proteomic assessment. Mole Cell Proteomics 2017; 16:824–39. 10.1074/mcp.M116.064733 PMC5417824 28265047

[24] Balbin A, Grubb A, Abrahamson M, Grubb R. Determination of allotypes G1m(f) and G1m(z) at the genomic level by subclass-specific amplification of DNA and use of allele-specific probes. Exp Clin Immunogenet 1991; 8:88–95. 1789994

[25] Webster CI, Bryson CJ, Cloake EA, et al A comparison of the ability of the human IgG1 allotypes G1m3 and G1m1,17 to stimulate T-cell responses from allotype matched and mismatched donors. MAbs 2016; 8:253–63. 26821574 10.1080/19420862.2015.1128605 PMC4966604

[26] Kyei-Baafour E, Kusi KA, Arthur FK, et al Association of immunoglobulin G3 hinge region length polymorphism with cerebral malaria in Ghanaian children. J Infect Dis 2022; 225:1786–90. 34718631 10.1093/infdis/jiab548

[27] Longley RJ, White MT, Takashima E, et al Development and validation of serological markers for detecting recent *Plasmodium vivax* infection. Nat Med 2020; 26:741–9. 32405064 10.1038/s41591-020-0841-4

[28] Pandey JP, Nasr A, Rocca KM, Troy-Blomberg M, Elghazali G. Significant differences in GM allotype frequencies between two sympatric tribes with markedly differential susceptibility to malaria. Parasite Immunol 2007; 29:267–9. 17430550 10.1111/j.1365-3024.2007.00938.x

[29] Andrews CA . Natural selection, genetic drift, and gene flow do not act in isolation in natural populations. Nat Educ Knowledge 2010; 3:5.

[30] Angrisano F, Robinson L. *Plasmodium vivax*—how hidden reservoirs hinder global malaria elimination. Parasitol Int 2022; 87:102526. 34896312 10.1016/j.parint.2021.102526

[31] Facer CA . Direct antiglobulin reactions in Gambian children with *P. falciparum* malaria III. Expression of IgG subclass determinants and genetic markers and association with anaemia. Clin Exp Immunol 1980; 41:81–90. 7002393 PMC1536921

[32] Fall AKDJ, Kana IH, Dechavanne C, et al Naturally acquired antibodies from Beninese infants promote *Plasmodium falciparum* merozoite-phagocytosis by human blood leukocytes: implications for control of asymptomatic malaria infections. Malar J 2022; 21:356. 36447200 10.1186/s12936-022-04361-w PMC9707106

[33] Morahan G, Berek C, Miller JFAP. An idiotypic determinant formed by both immunoglobulin constant and variable regions. Nature 1983; 301:720–2. 6186921 10.1038/301720a0

[34] Torres M, Fernández-Fuentes N, Fiser A, Casadevall A. The immunoglobulin heavy chain constant region affects kinetic and thermodynamic parameters of antibody variable region interactions with antigen. J Biol Chem 2007; 282:13917–27. 17353196 10.1074/jbc.M700661200

[35] Rivas-Fuentes S, Garcia-Garcia E, Nieto-Castaneda G, Rosales C. Fcγ receptors exhibit different phagocytosis potential in human neutrophils. Cell Immunol 2010; 263:114–21. 20356573 10.1016/j.cellimm.2010.03.006

[36] Feng G, Wines BD, Kurtovic L, et al Mechanisms and targets of Fcγ-receptor mediated immunity to malaria sporozoites. Nat Commun 2021; 12:1742. 33741975 10.1038/s41467-021-21998-4 PMC7979888

[37] Chu TH, Crowley AR, Backes I, et al Hinge length contributes to the phagocytic activity of HIV-specific IgG1 and IgG3 antibodies. PLoS Pathog 2020; 16:e1008083. 32092122 10.1371/journal.ppat.1008083 PMC7058349

[38] Steinberg AG, Morell A, Skvaril F, Van Loghem E. The effect of Gm (23) on the concentration of IgG2 and IgG4 in normal human serum. J Immunol 1973; 110:1642–5. 4712925

[39] Sarvas H, Rautonen N, Makela O. Allotype-associated differences in concentrations of human IgG subclasses. J Clin Immunol 1991; 11:39–45. 2022720 10.1007/BF00918793

[40] Pandey JP, French MA. GM phenotypes influence the concentrations of the four subclasses of immunoglobulin G in normal human serum. Hum Immunol 1996; 51:99–102. 8960912 10.1016/s0198-8859(96)00205-4

[41] Oxelius VA . Serum IgG and IgG subclass contents in different Gm phenotypes. Scand J Immunol 1993; 37:149–53. 8434227 10.1111/j.1365-3083.1993.tb01750.x

