# Supplementary material for: Association of a Novel IgG3 Allele With Malaria in Children From the Sepik Region of Papua New Guinea
Source: J Infect Dis. 2025 Jul 25;232(4):e555–64. doi: 10.1093/infdis/jiaf390 (PMC12526884; doi:10.1093/infdis/jiaf390)
Supplement: jiaf390_Supplementary_Data [file jiaf390_supplementary_data.docx]

***Journal of Infectious Diseases***

**MAJOR ARTICLE**

**Association of a novel IgG3 allele with malaria in children from the Sepik region of Papua New Guinea**

**Maria Saeed^1^, Elizabeth H Aitken^1,2^, Myo T Naung^3^, Caitlin Bourke^3^, Kenneth W Wu^3^, Rhea J Longley^3,4,5^, Amy W Chung^2^, Timon Damelang^2^, Benson Kiniboro^6^, Ivo Mueller^3,4^, Stephen J Rogerson*^1,7^**

^1^Department of Infectious Diseases, The Peter Doherty Institute for Infection and Immunity, University of Melbourne, Melbourne, Australia; ^2^Department of Microbiology and Immunology, The Peter Doherty Institute for Infection and Immunity, University of Melbourne, Melbourne, Australia; ^3^Infection and Global Health Division, Walter and Eliza Hall Institute, Parkville, Victoria, Australia; ^4^Department of Medical Biology, University of Melbourne, Melbourne, Australia; ^5^Faculty of Tropical Medicine, Mahidol University, Bangkok, Thailand; ^6^Vector Borne Disease unit, Papua New Guinea Institute of Medical Research, Goroka, Papua New Guinea; ^7^Department of Medicine, The Peter Doherty Institute for Infection and Immunity, University of Melbourne, Melbourne, Australia

*___________________________________________________________________________*

*Correspondence to: Prof Stephen Rogerson, The Peter Doherty Institute for Infection and Immunity, University of Melbourne, Melbourne, Australia. Email: [sroger@unimelb.edu.au](mailto:sroger@unimelb.edu.au)

**Supplementary Material**

| **Supplementary Table S1. Criteria for categorizing *Plasmodium* spp*.* infections** | |
| --- | --- |
| ***Plasmodium* spp. infections** | **criteria** |
| *P. vivax* asymptomatic infections | ≤500 parasites/μl with no febrile illness |
| *P. vivax* symptomatic infections | > 500 parasites/μl with febrile illness |
| *P. vivax* severe infections | > 500 parasites/μl, hemoglobin < 5g/dl with any danger signs (as per IMCI) |
| *P. falciparum* asymptomatic infections | ≤2500 parasites/μl with no febrile illness |
| *P. falciparum* symptomatic infections | > 2500 parasites/μl with febrile illness |
| *P. falciparum* hyperparasitemia | > 250,000 parasites/μl |

IMCI = Integrated Management of Childhood Illness

**Supplementary Table S2: Summary of *Plasmodium* spp*.* infections in children**

| ***Plasmodium* spp. infections** | **Number of episodes** |
| --- | --- |
| Total asymptomatic infections | 1791 |
| *P. vivax* asymptomatic infections | 1072 |
| *P. falciparum* asymptomatic infections | 719 |
| Total symptomatic infections | 734 |
| *P. vivax* symptomatic infections | 361 |
| *P. falciparum* symptomatic infections | 373 |
| *P. vivax* severe malaria infections | 27 |
| *P. vivax* severe malaria + *P. falciparum* hyperparasitemia | 35 |

**Supplementary Table S3: Primer sequences for *IGHG3* (C_H_2 + C_H_3), *IGHG1* (C_H_1 + C_H_3) and *IGHG3* hinge region.**

| **Gene** | **Primer sequences** | **References** |
| --- | --- | --- |
| IgG3  C_H_2 + C_H_3 | Forward primer 5’-GTCGGGTGCTGACACATCTG-3’  Reverse primer 5’-GCCCTGGACTGGGGCTGCAT-3’ | [23] |
| IgG1  C_H_1  C_H_3 | Forward primer 5’-CCCCTGGCACCCTCCTCCAA-3’  Reverse primer 5’-GCCCTGGACTGGGGCTGCAT-3’  Forward primer 5’-GAGCCCAAATCTTGTGACAA-3’  Reverse primer 5’-GGCGATGTCGCTGGGA-3’ | [24]  [25] |
| IgG3  Hinge | Forward primer 5’-AAAACCCCACTTGGTGACAC-3’  Reverse primer 5’-GGGTCCGGGAAATCATAAGG-3’ | [26] |

**Agarose gel electrophoresis**

Five μl of amplified PCR product was mixed with 1 μl of 6X DNA purple loading dye (New England Biolabs) and separated on a 2% agarose gel (Promega) in 1X trisaminomethane-acetate-ethylenediaminetetraacetic acid (TAE) buffer (Promega) with SyBR safe DNA dye.


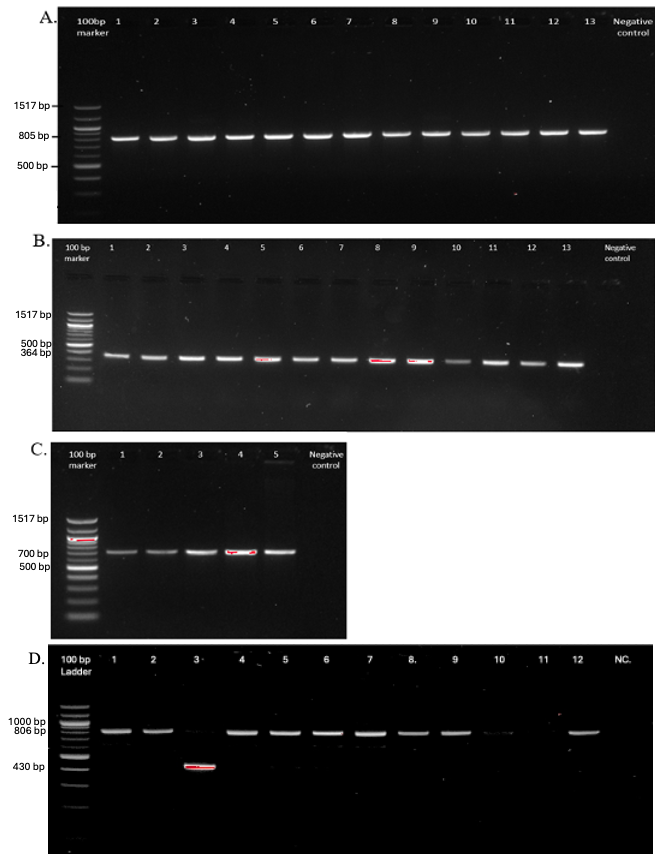


**Supplementary Figure S1. Agarose gel electrophoresis (2% agarose) for PCR amplified product using primers for IgG3-C_H_2+C_H_3, IgG1-C_H_1, IgG1-C_H_3 domains and IgG3 hinge region.** First lane has 100 bp marker and last well is a negative control. **(A)** Lane 1 to Lane 13 have amplified samples. All the samples have a band at 805 bp (C_H_2+C_H_3 domain), which confirmed amplification of IgG3 (C_H_2 + C_H_3). **(B)** All the samples in lane 1 to lane 13 have a 364 bp product (C_H_1 domain), which confirmed amplification of IgG1 C_H_1 domain. **(C)** Lane 1 to Lane 5 have the amplified samples and a 700 bp product (C_H_3 domain) **(D)** samples with long hinge (LL) 806 bp band and a sample with small hinge (SS) 430 bp in lane 3.

| Supplementary Table S4. Gene Dosage Effect of IGHG3*30 allele on *Plasmodium spp.* infections (*N=203*). | | | |
| --- | --- | --- | --- |
|  | **Coefficent** | **95% CI** | **p value** |
| *Plasmodium* spp. infections |  |  |  |
| IGHG3*30 heterozygous | -2.34 | [-4.13, -0.54] | **0.011** |
| IGHG3*30 homozygous | -1.27 | [-3.08, 0.54] | 0.169 |
| Asymptomatic *Plasmodium* spp. infections |  |  |  |
| IGHG3*30 heterozygous | -1.96 | [-3.28, -0.64] | **0.004** |
| IGHG3*30 homozygous | -1.25 | [-2.59, 0.07] | 0.065 |
| *P. vivax* asymptomatic infections |  |  |  |
| IGHG3*30 heterozygous | -1.47 | [-2.49, -0.44] | **0.005** |
| IGHG3*30 homozygous | -0.74 | [-1.78, 0.29] | 0.157 |
| *P. falciparum* asymptomatic infections |  |  |  |
| IGHG3*30 heterozygous | -0.45 | [-1.36, 0.45] | 0.325 |
| IGHG3*30 homozygous | -0.17 | [-1.10, 0.74] | 0.705 |
| Symptomatic *Plasmodium* spp. infections |  |  |  |
| IGHG3*30 heterozygous | -0.39 | [-1.40, 0.42] | 0.388 |
| IGHG3*30 homozygous | -0.35 | [-1.41, 0.43] | 0.444 |
| *P. vivax* symptomatic infections |  |  |  |
| IGHG3*30 heterozygous | -0.04 | [-0.76, 0.68] | 0.913 |
| IGHG3*30 homozygous | 0.22 | [-0.51, 0.95] | 0.550 |
| *P. falciparum* symptomatic infections |  |  |  |
| IGHG3*30 heterozygous | -0.32 | [-0.96, 0.31] | 0.323 |
| IGHG3*30 homozygous | -0.56 | [-1.21, 0.08] | 0.086 |
| *P. vivax* severe malaria infections |  |  |  |
| IGHG3*30 heterozygous | -0.11 | [-0.23, 0.01] | 0.070 |
| IGHG3*30 homozygous | -0.05 | [-0.18, 0.07] | 0.387 |
| *P. vivax* severe + *P. falciparum* hyperparasitemia |  |  |  |
| IGHG3*30 heterozygous | -0.12 | [-0.26, 0.01] | 0.082 |
| IGHG3*30 homozygous | -0.06 | [-0.20, 0.08] | 0.397 |
| Non-IGHG3*30 was the reference category for each analysis. | | | |

| Supplementary Table S5: Association between IGHG1*01 and *Plasmodium* spp. infections (*N=200*). | | | |
| --- | --- | --- | --- |
|  | **Coefficient** | **95% CI** | **p value** |
| *Plasmodium* spp. infections | -1.95 | [-3.88, -0.02] | **0.047** |
| Asymptomatic *Plasmodium* spp. infections | -1.22 | [-2.65, 0.21] | 0.096 |
| *P. vivax* asymptomatic infections | -0.51 | [-1.62, 0.61] | 0.371 |
| *P. falciparum* asymptomatic infections | -0.72 | [-1.69, 0.23] | 0.139 |
| Symptomatic *Plasmodium* spp. infections | -0.56 | [-1.53, 0.40] | 0.254 |
| *P. vivax* symptomatic infections | -0.19 | [-0.96, 0.58] | 0.629 |
| *P. falciparum* symptomatic infections | -0.35 | [-1.04, 0.33] | 0.309 |
| *P. vivax* severe malaria infections | -0.11 | [-0.24, 0.02] | 0.089 |
| *P. vivax* severe + *P. falciparum* hyperparasitemia | -0.12 | [-0.27, 0.02] | 0.103 |

IGHG1*01 homozygous was the reference category.

| Supplementary Table S6. Linkage between IGHG3*30 and IGHG1*01 | | | |
| --- | --- | --- | --- |
| **IGHG3 allele** | **IGHG1*01 Homozygous** | **IGHG1*01 Heterozygous** | **Total** |
| IGHG3*30 Homozygous | 71 (41.7%) | 5 (16.6 %) | 76 |
| IGHG3*30 Heterozygous | 62 (36.5%) | 19 (63.3%) | 81 |
| Non-IGHG3*30 | 37 (21.8%) | 6 (20.0%) | 43 |
| Total | 170 | 30 | 200 |

*X^2^* (2, *N*=200) = 8.81 *p =* 0.012


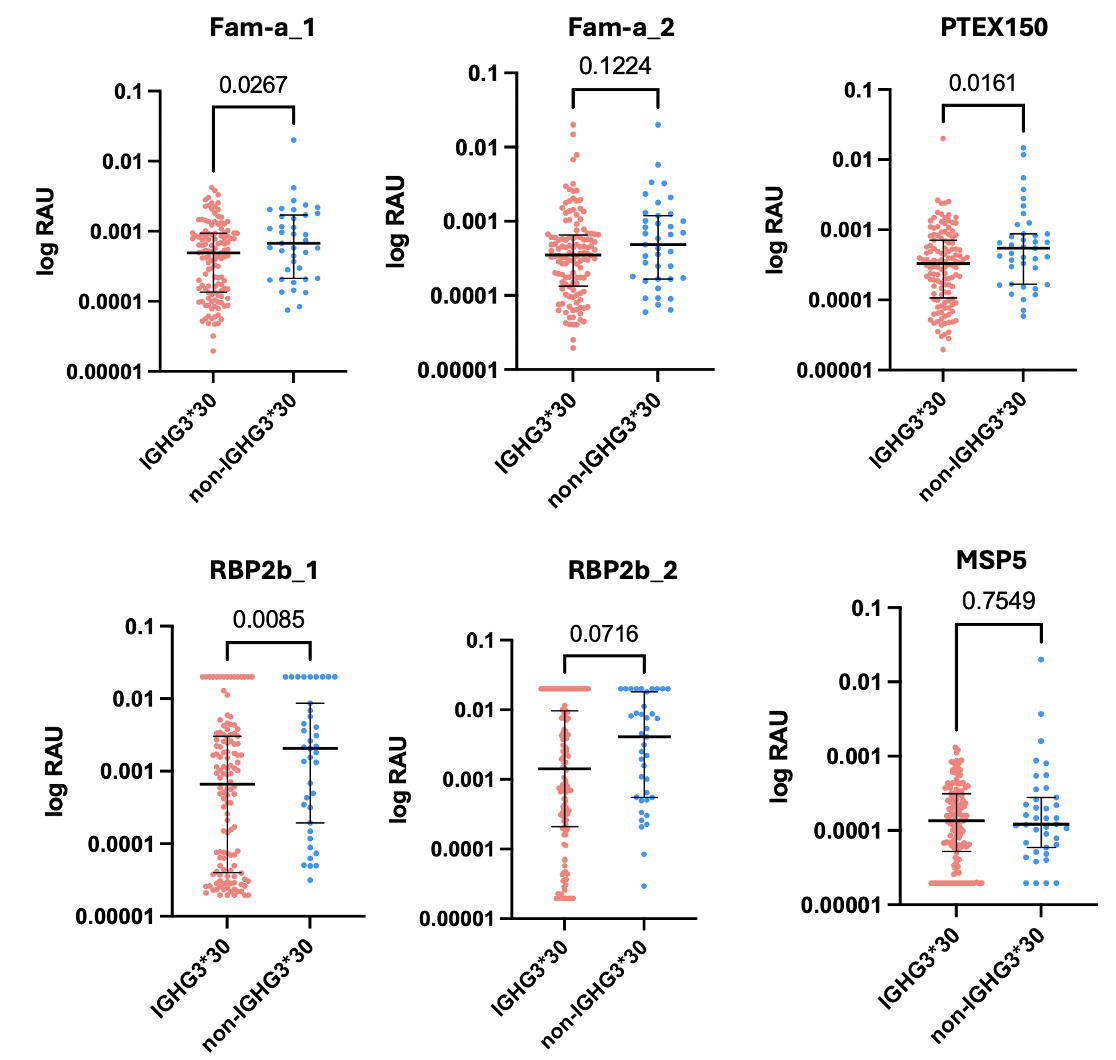


**Supplementary Figure S2. Total IgG levels to six *P. vivax* proteins in IGHG3*30 allele carriers and non-IGHG3*30 allele carriers (*N=167*).** Data is presented as median and interquartile range (IQR). Y-axis show log transformed RAU values. *P* values were determined by Mann-Whitney U-test. RAU; relative antibody unit

| Supplementary Table S7: Association between IGHG3*30 allele and *Plasmodium spp.* infections (adjusted for IgG levels) (*N=167).* | | | | |
| --- | --- | --- | --- | --- |
|  | **Unadjusted β**  **(95% CI)** | **p-value** | **Adjusted β**  **(95% CI)** | **p-value** |
| *Plasmodium* spp. infections | -1.82 (-3.55, -0.11) | 0.038 | 1.82 (-3.58, -0.06) | **0.027** |
| Asymptomatic *Plasmodium* spp. infections | -1.74 (-3.02, -0.47) | **0.007** | -1.74 (-2.97, -0.51) | **0.006** |
| *P. vivax* asymptomatic infections | -1.37 (-2.34, -0.41) | **0.006** | -1.36 (-2.28, -0.44) | **0.004** |
| *P. falciparum* asymptomatic infections | -0.28 (-1.16, 0.61) | 0.530 | -0.28 (-1.11, 0.55) | 0.508 |
| Symptomatic *Plasmodium* spp. infections | -0.25 (-1.14, 0.64) | 0.580 | -0.52 (-1.15, 0.65) | 0.580 |
| *P. vivax* symptomatic infections | 0.21 (-0.48, 0.91) | 0.540 | 0.21 (-0.46, 0.89) | 0.530 |
| *P. falciparum* symptomatic infections | -0.38 (-1.01, 0.24) | 0.230 | -0.38 (-0.97, 0.21) | 0.202 |
| *P. vivax* severe malaria infections | 0.04 (-0.16, 0.07) | 0.490 | 0.04 (-0.16, 0.08) | 0.480 |
| *P. vivax* severe + *P. falciparum* hyperparasitemia | -0.04 (-0.18, 0.09) | 0.520 | -0.04 (-0.14, 0.14) | 0.520 |

Non-IGHG3*30 was the reference category for each analysis.

Multiple linear regression was performed between IGHG3*30 allele carriage and *Plasmodium spp.* infections after adjusting for IgG levels to six *P. vivax* proteins (divided into tertiles; low, medium, high).
